# Thyroid nodule segmentation in ultrasound images using transformer models with masked autoencoder pre-training

**DOI:** 10.3389/frai.2025.1618426

**Published:** 2025-07-24

**Authors:** Yi Xiang, Rajendra Acharya, Quan Le, Jen Hong Tan, Chiaw-Ling Chng

**Affiliations:** ^1^Office of Insights & Analytics, Division of Digital Strategy, SingHealth, Singapore, Singapore; ^2^School of Mathematics, Physics and Computing, University of Southern Queensland, Springfield Central, QLD, Australia; ^3^Data Science and Artificial Intelligence Lab, Singapore General Hospital, Singapore, Singapore; ^4^Department of Endocrinology, Singapore General Hospital, Singapore, Singapore

**Keywords:** thyroid nodule segmentation, ultrasound imaging, transformer-based network, Masked Autoencoder, self-supervised learning

## Abstract

**Introduction:**

Thyroid nodule segmentation in ultrasound (US) images is a valuable yet challenging task, playing a critical role in diagnosing thyroid cancer. The difficulty arises from factors such as the absence of prior knowledge about the thyroid region, low contrast between anatomical structures, and speckle noise, all of which obscure boundary detection and introduce variability in nodule appearance across different images.

**Methods:**

To address these challenges, we propose a transformer-based model for thyroid nodule segmentation. Unlike traditional convolutional neural networks (CNNs), transformers capture global context from the first layer, enabling more comprehensive image representation, which is crucial for identifying subtle nodule boundaries. In this study, We first pre-train a Masked Autoencoder (MAE) to reconstruct masked patches, then fine-tune on thyroid US data, and further explore a cross-attention mechanism to enhance information flow between encoder and decoder.

**Results:**

Our experiments on the public AIMI, TN3K, and DDTI datasets show that MAE pre-training accelerates convergence. However, overall improvements are modest: the model achieves Dice Similarity Coefficient (DSC) scores of 0.63, 0.64, and 0.65 on AIMI, TN3K, and DDTI, respectively, highlighting limitations under small-sample conditions. Furthermore, adding cross-attention did not yield consistent gains, suggesting that data volume and diversity may be more critical than additional architectural complexity.

**Discussion:**

MAE pre-training notably reduces training time and helps themodel learn transferable features, yet overall accuracy remains constrained by limited data and nodule variability. Future work will focus on scaling up data, pre-training cross-attention layers, and exploring hybrid architectures to further boost segmentation performance.

## 1 Introduction

Thyroid nodules are commonly found in the general population and are often detected through imaging, either during investigations for thyroid-related issues or as incidental findings (Dean and Gharib, 2008). While most nodules are benign and asymptomatic, a small percentage can be malignant, requiring timely and accurate evaluation. Segmentation is an essential initial step in this process, as it delineates the interface between the nodule and the surrounding parenchyma, aiding in the assessment of malignancy likelihood.

Segmenting thyroid nodules in ultrasound images presents several technical challenges. The lack of distinct anatomical landmarks, coupled with the low contrast between tissues, makes it difficult to differentiate the boundaries of the nodules. Additionally, the granular speckle noise inherent in ultrasound imaging adds further complexity by distorting image clarity and increasing the variability of nodule shapes and appearances across frames.

Early segmentation methods, such as K-means (Hart et al., 2000), Fuzzy C-means (FCM) (Hart et al., 2000), efficient graph-based segmentation (EGB) (Felzenszwalb and Huttenlocher, 2004), and robust graph-based segmentation (RGB) (Huang et al., 2012), were among the earliest techniques applied in computer-aided image segmentation. These methods rely on pre-defined parameters, such as thresholds, which are manually crafted and need careful tuning for optimal performance (Xu et al., 2019). Despite their simplicity and effectiveness in certain cases, these methods often struggle to adapt to complex patterns in large and diverse datasets.

With the advancement of data availability and computational power, deep learning-based approaches, particularly convolutional neural networks (CNNs), have gained prominence. CNNs excel at capturing patterns between inputs and outputs by learning features directly from data, eliminating the need for handcrafted features. Traditional CNNs for segmentation often utilize an encoder-decoder architecture. The encoder extracts low-resolution feature maps, while the decoder up-samples these maps to produce per-pixel class predictions. Fully Convolutional Networks (FCNs) (Long et al., 2015) represent a classic implementation of this architecture. Building on this, models such as U-Net (Ronneberger et al., 2015) introduced skip connections between the encoder and decoder, allowing the combination of fine-grained details from earlier layers with deeper, more abstract features, thus preserving critical spatial information.

However, one key limitation of CNNs is their inability to effectively capture global image context due to the local nature of convolutional filters. Although deeper layers in CNNs can expand the receptive field, they still struggle to form a comprehensive view of the entire image, making it difficult to accurately segment structures like thyroid nodules. Transformers (Vaswani, 2017), in contrast, inherently capture global context from the very first layer, offering a more holistic image representation that is crucial for detecting subtle boundary variations. However, despite their strengths, Vision Transformers (ViTs) tend to underperform on smaller datasets compared to CNNs, as they lack the inductive biases that help CNNs generalize well with limited data (Dosovitskiy, 2020).

In this study, we deviate from the typical approach of pre-training ViTs on classification datasets like ImageNet-21k. Instead, we aim to leverage the segmentation dataset more effectively by pre-training the model using a Masked Autoencoder (MAE) (He et al., 2022). The MAE reconstructs partially masked images, enabling the model to capture image patterns more effectively for segmentation tasks.

We perform an extensive analysis of transformer architectures for segmentation, experimenting with different model architectures and input patch sizes. Inspired by advancements in natural language processing (Vaswani, 2017), we incorporate a cross-attention mechanism to improve the interaction between the encoder and decoder, enhancing the model's context capture capabilities.

In summary, we propose a transformer-based approach with MAE pre-training for thyroid nodule segmentation, performing ablation studies on model architectures and patch sizes to optimize performance for this challenging task.

## 2 Methods

### 2.1 Dataset

For the pre-training and segmentation tasks, we utilize three open-source datasets: AIMI, TN3K (Gong et al., 2021), and DDTI (Pedraza et al., 2015). The AIMI dataset was collected from 167 patients with 192 biopsy-confirmed thyroid nodules at the Stanford University Medical Center. The TN3K dataset consists of ultrasound images provided in Gong et al. (2021), while the DDTI dataset was compiled with the support of the Universidad Nacional de Colombia. The specifics of each dataset, including the number of images and the corresponding patient data, are outlined in Table 1.

**Table 1:** Dataset and splitting details.

**Dataset**	**Total number of images**	**Training images**	**Testing images**
AIMI	17,412 (from 192 subjects)	14,055 (from 154 subjects)	3,357 (from 38 subjects)
TN3K	3,493	2,879	614
DDTI	637	477	160

For model training and evaluation, we split each dataset into training and testing subsets. The AIMI and TN3K datasets were split in an 80:20 ratio, while the DDTI dataset was split in a 75:25 ratio. The exact number of images used for training and testing across each dataset is presented in Table 1.

We applied these splits in two key stages of the process:

- For MAE pre-training, we used only the training portion of each dataset to pre-train the model.
- For the segmentation model, we trained the model on the training subset and evaluated its performance on the testing subset to assess generalization capabilities.

### 2.2 Masked autoencoder (MAE)

We follow the self-supervised training framework outlined in the original MAE paper (He et al., 2022), utilizing an encoder-decoder architecture to pre-train a Vision Transformer (ViT). In this process, the input ultrasound images are first resize to 224 × 224 and then divided into non-overlapping patches of size 14 × 14. We randomly mask 75% of the patches, as recommended in original MAE paper.

The remaining 25% of the unmasked patches are fed into the encoder, a 12-layer ViT with a patch embedding size of 192 and three attention heads. The masked patches are not passed through the encoder but are later included in the decoder stage. The decoder, consisting of a four-layer ViT with three attention heads, receives both the encoded unmasked patches and a learned representation for the masked patches. The decoder then reconstructs the entire image, and a linear projection layer maps the output back to the original image resolution.

In the original MAE framework, reconstruction loss is measured using mean squared error (MSE) on the masked patches. We extend this by incorporating loss from the unmasked patches as well to enhance the model's attention to both masked and visible regions. Our modified loss function is:


$$\begin{array}{l} Loss=MSE_{masked\text{\_}patches}+\alpha MSE_{unmasked\text{\_}patches} \end{array}$$

where α is a weighting factor that balances the contribution of the unmasked patches to the total loss. We empirically set α = 0.1 to encourage focus on the reconstruction of masked regions while still accounting for some information from unmasked patches.

We train the MAE using the AdamW optimizer with an initial learning rate of 2 × 10^−5^, a batch size of 1.024, and 4.000 epochs. Data augmentation techniques, including random horizontal flips and random resized crops, are applied to increase the diversity of training data and improve the model's generalizability, especially given the relatively small size of the thyroid nodule dataset.

### 2.3 Segmentation model architecture

The segmentation model largely follows the same structure as the MAE pre-training process, with key differences in how all image patches are processed. After resizing and dividing the input ultrasound images into non-overlapping patches, all patches are passed through the encoder and decoder layers. We maintain the similar architecture as in the MAE process, using a 12-layer encoder and a three-layer decoder, to facilitate the comparison between the model trained from scratch and the one fine-tuned with MAE pre-trained weights.

**Figure 1:**
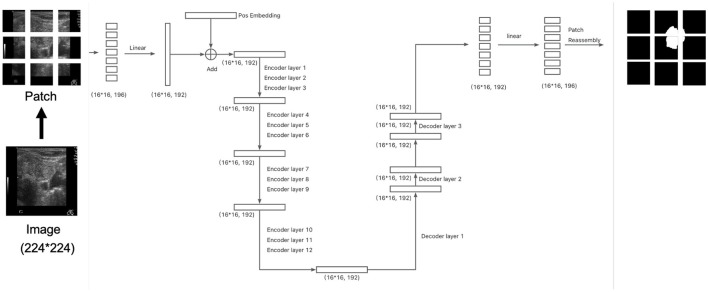
The architecture of the segmentation model. The division of patches at here is for illustration. Please refer to section 2.3 for actual setup.

Specifically, the input images are resized to (224 × 224) pixels and divided into (14 × 14) patches, resulting in 256 patches per image. After flattening each patch, a linear layer projects the resulting vectors into a 192-dimensional space. Positional embeddings are then added, resulting in an input tensor of shape (16 × 16, 192). This tensor is passed through the 12-layer encoder followed by the three-layer decoder. The final output of the decoder is projected to 196 dimensions (corresponding to the flattened segmentation map) and then passed through a sigmoid activation function to generate the segmentation mask, as illustrated in Figure 1. The total number of parameters in this configuration is 4.66M.

We train the segmentation model for 400 epochs using an initial learning rate of 1.6 × 10^−4^ and a batch size of 512. The Dice Loss is employed as the loss function to directly optimize for segmentation performance. Data augmentation techniques, including random rotation, random horizontal flips, and random resized cropping, are applied to enhance model generalization, especially given the limited size of the training dataset.

### 2.4 Cross-attention architecture

In addition to the traditional encoder-decoder architecture using Vision Transformer (ViT) layers, we also explore a cross-attention mechanism within the segmentation model. Cross-attention, originally introduced in natural language processing (NLP) (Vaswani, 2017), connects the encoder and decoder by allowing information exchange between the two, rather than relying solely on self-attention. This enhances the model's ability to leverage feature representations at multiple levels, similar to U-Net's skip connections.

**Figure 2:**
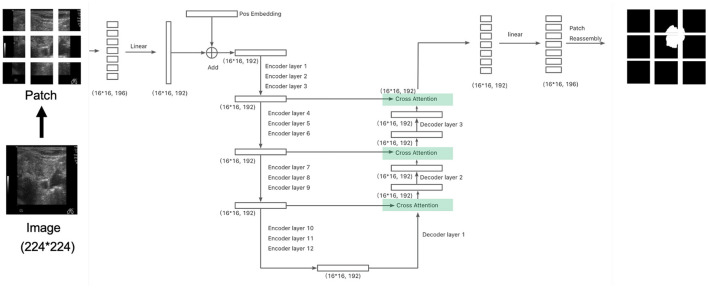
The architect of the segmentation model with cross attention: connecting the encoder and decoder similar to a U-net network. The division of patches at here is for illustration. Please refer to section 2.4 for actual setup.

The architecture we implemented is illustrated in Figure 2. In each cross-attention layer, the attention mechanism is computed as:


(1) $$\begin{array}{l} Attention\left(Q,K,V\right)=softmax\left(\frac{Q@K^{T}}{\sqrt{d_{k}}}\right)V \end{array}$$

In this case, the query (*Q*) comes from the decoder, while the key (*K*) and value (*V*) are sourced from the encoder output. This differs from the self-attention mechanism, where the query, key, and value all originate from the same source (either the encoder or decoder). Cross-attention allows the model to focus on relevant regions in the encoder output while processing the decoder's output, effectively linking the two stages. After incorporating the cross-attention layer, the total number of parameters increases to 5.54M, representing a 18.88% increase compared to the architecture without cross-attention.

This type of cross-attention architecture is widely used in NLP, most notably in models like T5 (Raffel et al., 2020), which employs an encoder-decoder structure with cross-attention to enhance information flow between the two components.

For our segmentation task, we designed a cross-attention-based architecture similar to U-Net, adding skip connections from the encoder to the decoder. This modification aims to better preserve spatial details and contextual information during the decoding process. We compare the performance of this cross-attention architecture with the original segmentation architecture in the following experiments to assess the impact of this design on segmentation accuracy and boundary detection.

## 3 Results

### 3.1 Masked autoencoder (MAE)

**Figure 3:**
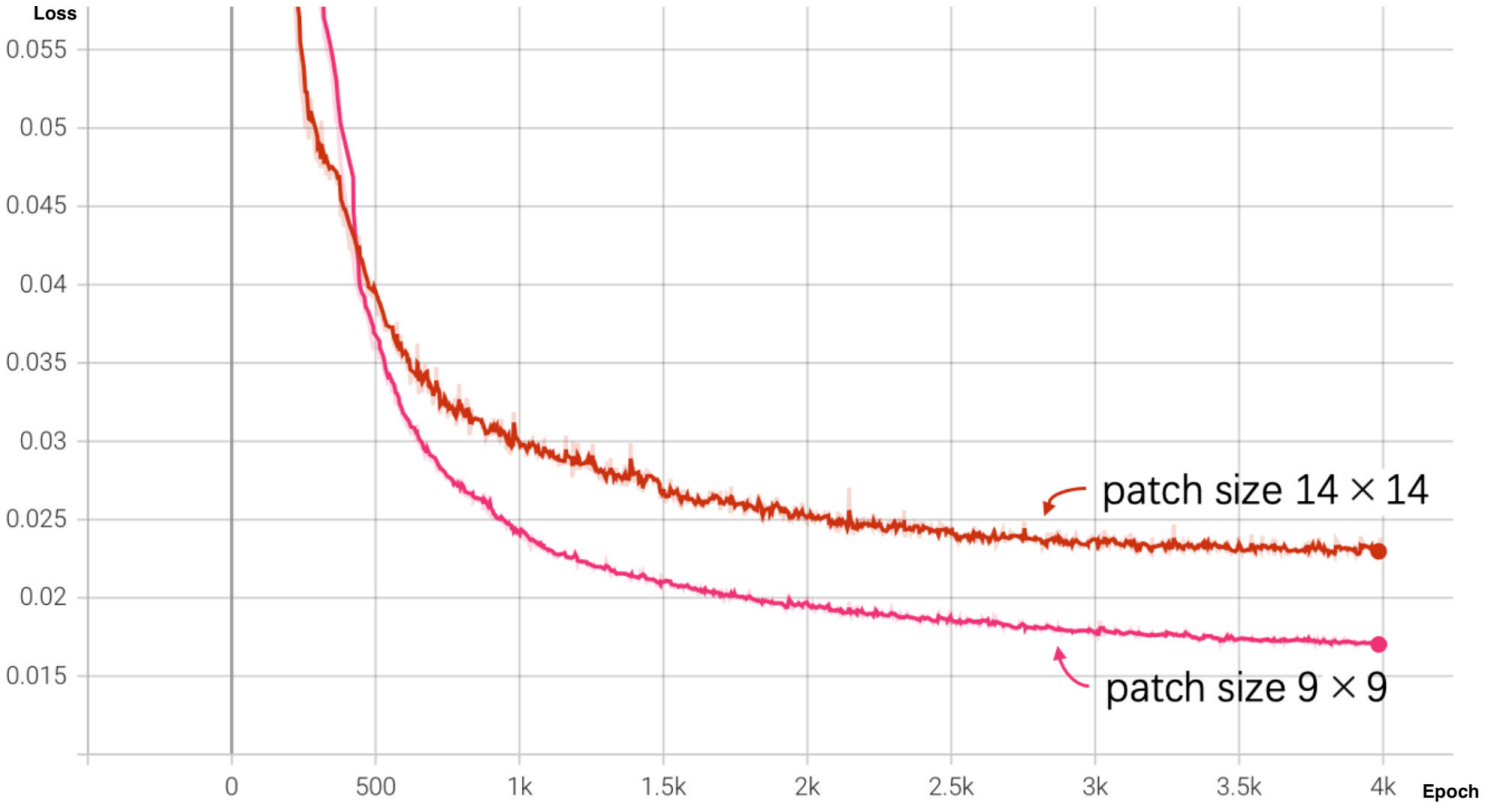
MAE loss curves for different patch sizes over 4,000 epochs. The pink curve represents the loss for patch size 9 × 9, and the red curve represents the loss for patch size 14 × 14.

We pre-trained the MAE model using the training dataset (size = 17,411) over 4,000 epochs. To evaluate the influence of patch size, we experimented with two configurations: a smaller patch size of 9 × 9 and a larger patch size of 14 × 14. The loss curves for both configurations are shown in Figure 3.

At the end of training (epoch 4,000), the MAE with a patch size of 9 × 9 achieved a lower reconstruction loss of 0.01698 compared to 0.02311 for the patch size of 14 × 14. As illustrated by the graph, the loss for patch size 9 decreased more rapidly during the initial training stages and outperformed patch size 14 throughout the training process, indicating that smaller patches facilitate better reconstruction performance.

**Figure 4:**
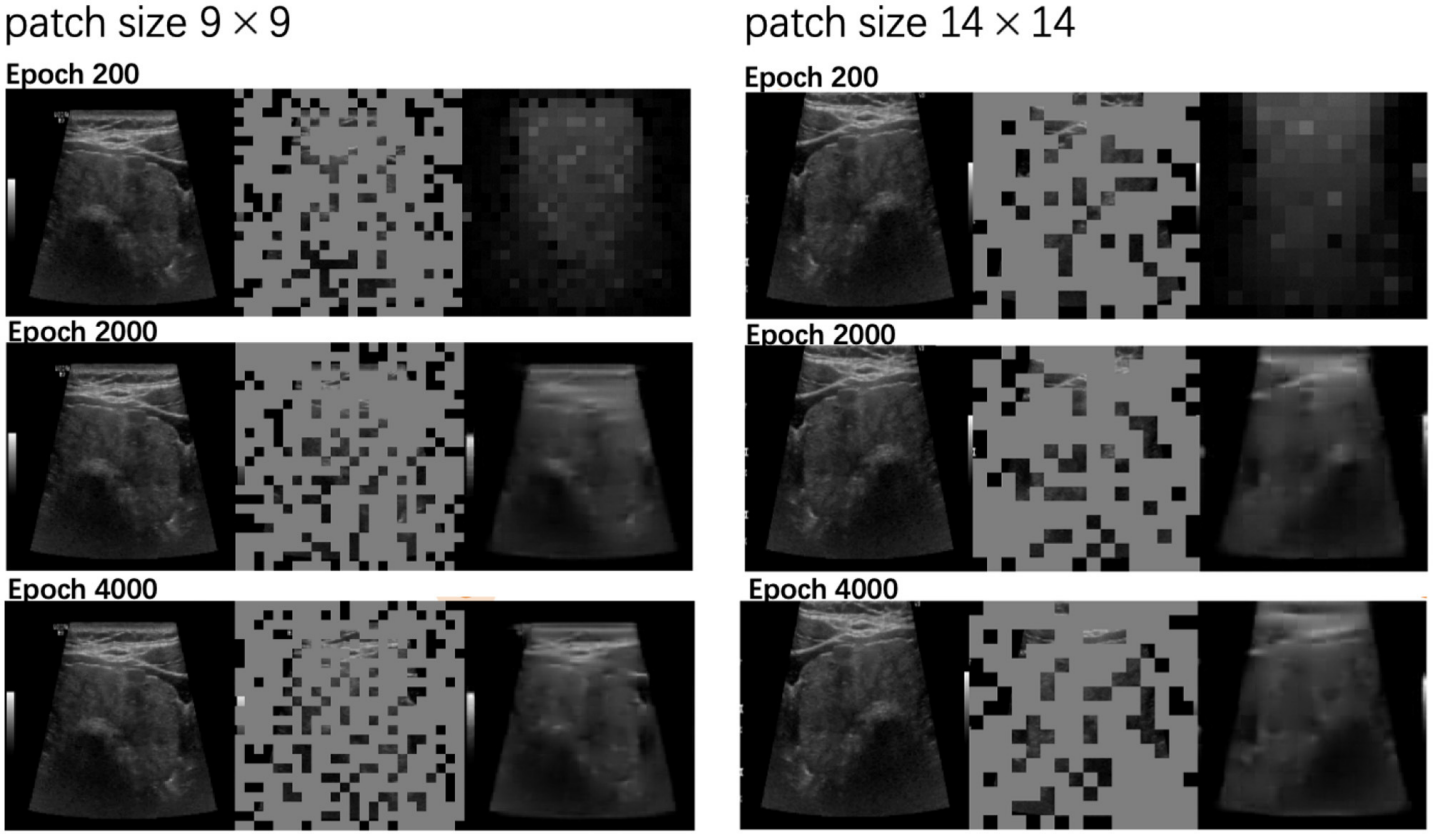
Reconstructed outputs by MAE with patch sizes 9 × 9 and 14 × 14 at different training stages (epochs 200, 2000, 4000). For each triplet, the original image is on the left, the masked input is in the middle, and the MAE-reconstructed image is on the right.

We further evaluated the quality of the reconstructed images at various training stages: epochs 200, 2,000, and 4,000. Figure 4 demonstrate the evolution of the reconstructed outputs for both patch sizes. The results show that as training progresses, the quality of the reconstructions improves significantly for both patch sizes.

Figure 4 also compares the reconstruction performance of the two patch sizes. The left column displays results for patch size 9 × 9, while the right column corresponds to patch size 14 × 14. Notably, the 9 × 9 patch size produced more detailed reconstructions, successfully capturing finer image features compared to the 14 × 14 patch size, which led to slightly coarser results. This suggests that smaller patch sizes enable the model to learn and preserve more intricate details during the reconstruction process.

### 3.2 Segmentation models

#### 3.2.1 Model performance pretrained with MAE vs. without

Given the architectural similarity between the encoder-decoder structure in MAE and the segmentation model, we trained the segmentation model both from scratch and using weights pretrained from the MAE process. Since the MAE decoder has four layers, while the segmentation model's decoder consists of three layers, we dropped the last layer of the MAE model when loading the weights.

**Figure 5:**
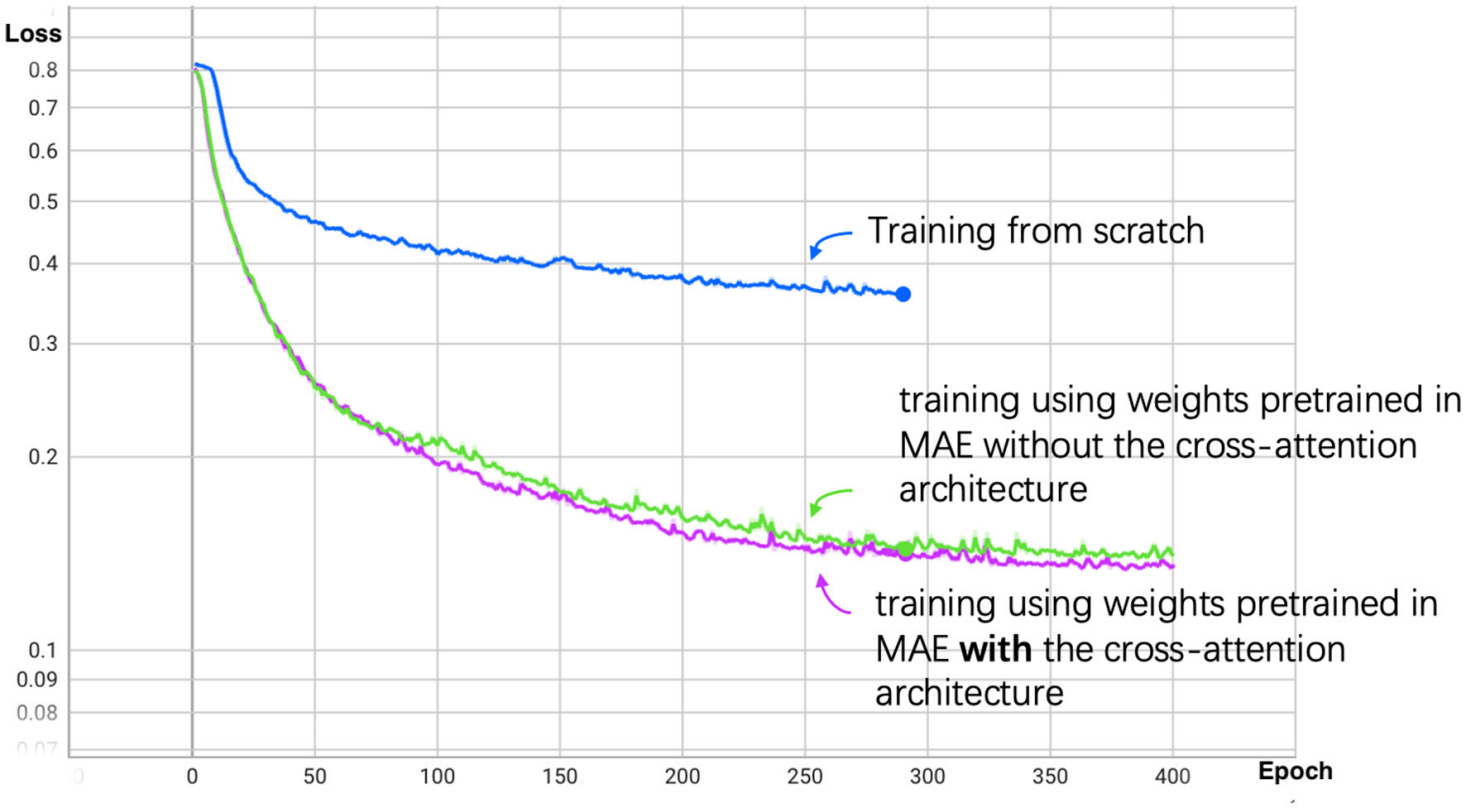
Segmentation loss curve for patch size 14. The blue line represents the training process from scratch, the green line represents training using weights pretrained in MAE without the cross-attention architecture, and the purple line represents training with cross-attention architecture.

We set the total training epochs to 400. As shown in Figure 5, the loss curve for training from scratch decreases slowly, leading us to apply early stopping. Subsequent experiments use the segmentation model initialized with the pretrained MAE weights.

#### 3.2.2 Performance with different architectures and patch sizes

As demonstrated in the previous section, the pretraining process significantly improved performance and reduced training time. We further compared different model architectures and patch sizes after pretraining.

The Dice score performance of our transformer-based segmentation model is shown in Table 2, alongside results from baseline counterparts, including UNet (Ronneberger et al., 2015), Attention UNet (Oktay et al., 2018), SResUNet-AD (Radhachandran et al., 2024), BPAT-UNet (Bi et al., 2023), UNet Transformer (Petit et al., 2021), and TransUNet (Chen et al., 2021). While the data splitting methods for these baselines are not entirely identical, the results remain comparable. Our model demonstrates notable improvements over SResUNet-AD, which primarily excels at reducing false positives; this advantage may be less relevant in the AIMI and DDTI datasets, as they exclusively include nodule images. However, our model's transformer-based architecture, without any convolutional neural network (CNN) layers, may explain its inferior performance compared to CNN-based models or hybrid approaches that integrate CNN and transformer architectures.

**Table 2:** Dice score comparison of segmentation models across datasets and patch sizes.

	**Dice score**		
	**AIMI**	**TN3K**	**DDTI**
UNet (Ronneberger et al., 2015)	0.7003	0.7998	0.6983
Attention UNet (Oktay et al., 2018)	**0.7129**	0.8114	0.7105
SResUNet-AD (Radhachandran et al., 2024)	0.5920 ± 0.369	–	0.4020 ± 0.384
BPAT-UNet (Bi et al., 2023)	–	**0.8364**	–
Unet Transformer (Petit et al., 2021)	–	0.8080	–
TransUNet (Chen et al., 2021)	–	0.8098	**0.8350**
With-cross attention-9 × 9	0.6254	0.6173	0.6537
Without-cross attention-9 × 9	0.6182	0.6342	0.6555
With-cross attention-14 × 14	0.6304	0.6390	0.6653
Without-cross attention-14 × 14	0.6321	0.6354	0.6479

We evaluated two patch sizes, 9 × 9 and 14 × 14, and two architectures: one with cross-attention (Figure 2) and one without cross-attention (Figure 1). The Dice scores for different datasets are reported in Table 2. Overall, the performance difference between models with and without cross-attention was not substantial, though there were some minor improvements in specific datasets.

**Figure 6:**
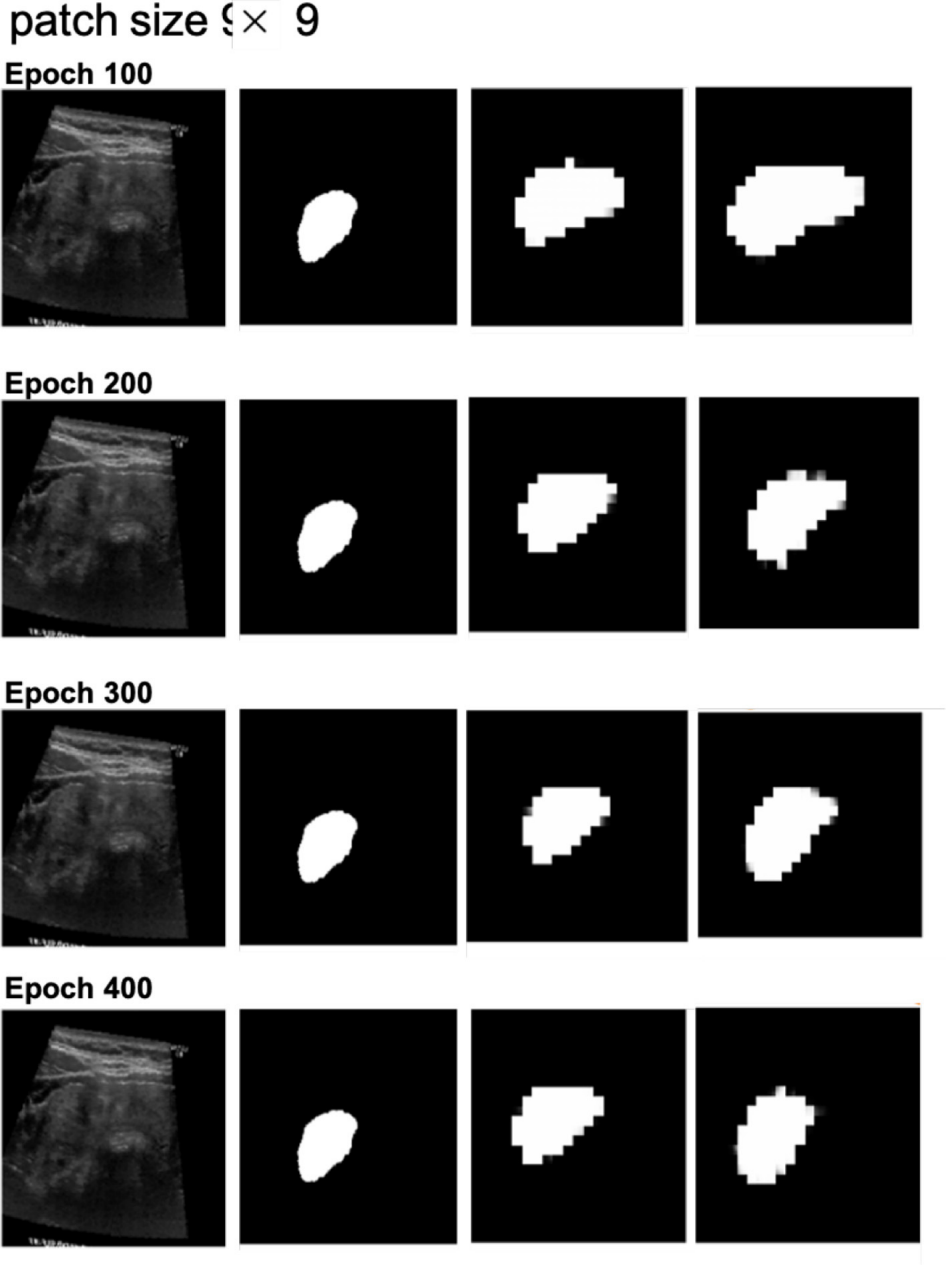
Segmentation results at different epochs (100, 200, 300, and 400) for patch size 9 × 9 with and without cross-attention. Each quadruplet includes the original ultrasound image, the gold standard, the predicted mask using the cross-attention model, and the predicted mask using the model without cross-attention.

**Figure 7:**
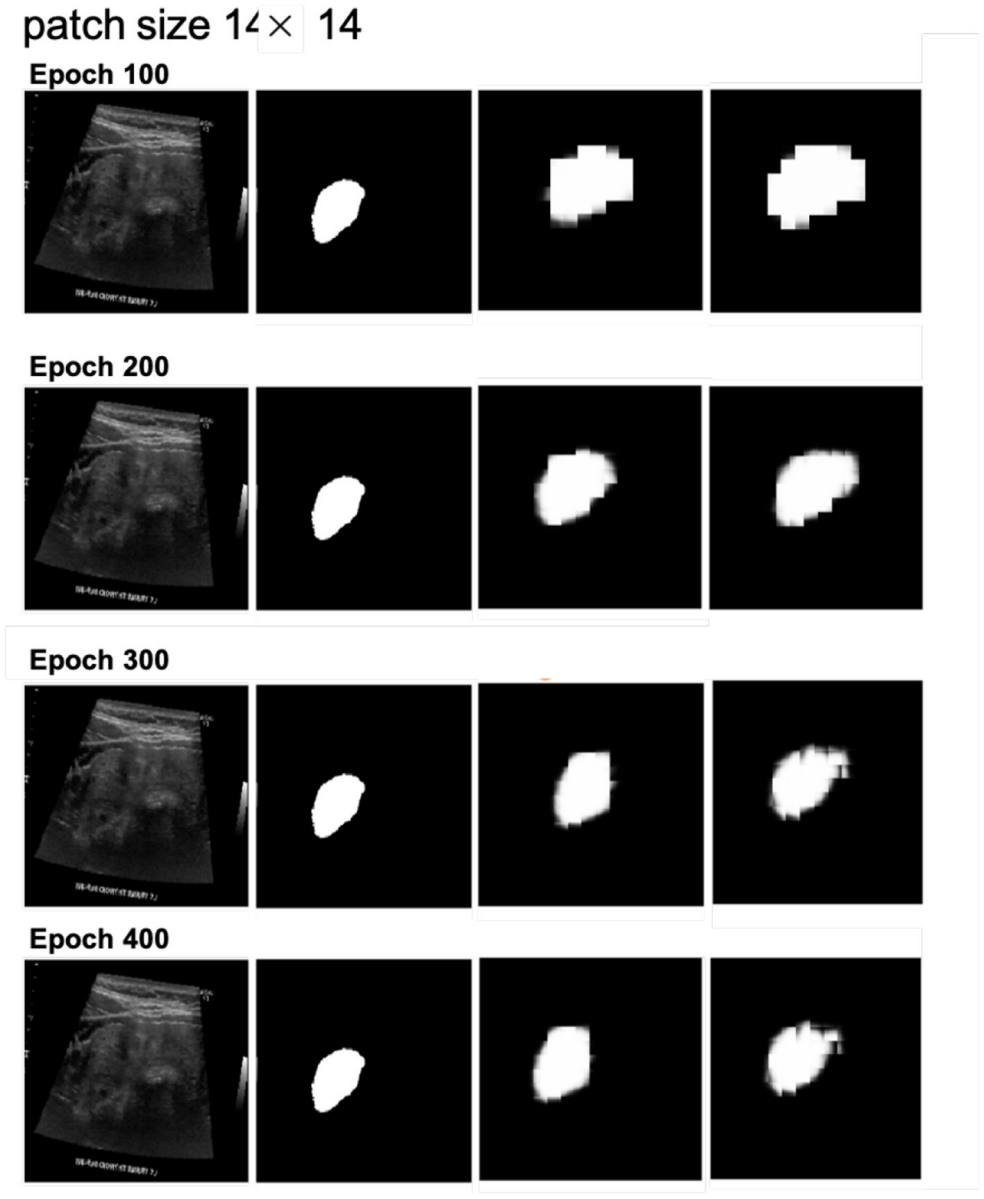
Segmentation results at different epochs (100, 200, 300, and 400) for patch size 14 × 14 with and without cross-attention. Each quadruplet includes the original ultrasound image, the gold standard, the predicted mask using the cross-attention model, and the predicted mask using the model without cross-attention.

Next, we examined the segmentation results. Figures 6, 7 show sample images from the training dataset, presenting segmentation results at epochs 100, 200, 300, and 400. Over the training process, the margins evolved from showing a significant mosaic effect to having smoother boundaries. While patch size 9 × 9 suffered from a more pronounced mosaic effect, it achieved higher positional accuracy compared to patch size 14 × 14. The difference in results between models with and without cross-attention was minor.

## 4 Discussion

The results demonstrate that using Masked Autoencoder (MAE) pretraining significantly improves the efficiency of the segmentation model training process. By initializing the segmentation model with weights pre-trained on the MAE task, we were able to achieve faster convergence compared to training the segmentation model from scratch. This suggests that MAE effectively transfers useful features, allowing the segmentation model to fully utilize the available training data and reduce overall training time.

To further analyze the proposed method, we compare its advantages and limitations with those of traditional CNN-based approaches and hybrid CNN-Transformer architectures. Table 3 summarizes the comparison.

**Table 3:** Advantages and limitations of different methods.

**Method**	**Advantages**	**Limitations**
Traditional CNNs (e.g., U-Net, Attention U-Net, SResUNet-AD)	- Effectively captures complex patterns between inputs and outputs. - Well-established and widely used in medical imaging.	- Limited ability to capture global context due to local receptive fields. - Requires large-scale pretraining datasets (e.g., ImageNet).
Hybrid CNN-Transformer Networks (e.g., BPAT-UNet, UNet transformer, TransUNet)	- Combines CNN's strength in extracting local features with Transformer's global context understanding. - High accuracy and strong performance metrics reported in various studies.	- Computationally expensive. - Often tuned for specific tasks and datasets, which may limit generalization for new applications.
Pure transformer networks with masked autoencoder pretraining(this study)	- Possesses the ability to capture the global context. - Masked Autoencoder (MAE) pretraining improves training efficiency by reducing training time and fully utilizing the dataset. - Eliminates the need for external datasets for pretraining.	- Mosaic artifacts in results, particularly with small patch sizes. - Moderate segmentation accuracy and dice scores compared to hybrid methods.

Traditional CNN-based methods, such as U-Net and Attention U-Net, excel at extracting local features and benefit from extensive pretraining on datasets like ImageNet. However, their limited receptive fields constrain their ability to capture global context, making them less effective for tasks requiring fine-grained boundary detection, such as thyroid nodule segmentation. Hybrid CNN-Transformer architectures, such as BPAT-UNet and TransUNet, leverage the strengths of both CNNs and Transformers, achieving high performance metrics. Nevertheless, these models are computationally expensive and often require careful tuning for specific datasets.

The proposed method, based on pure Transformer architecture with MAE pretraining, addresses some of these challenges by capturing global context and improving training efficiency. However, as shown in the results, the incorporation of the cross-attention mechanism did not lead to significant improvements. This could be due to the lack of pretraining for the cross-attention layers, which limits their effectiveness given the constraints of the training data. Future work could explore methods to pre-train these layers or leverage larger datasets to enhance their potential.

In terms of patch size, the smaller patch size (9 × 9) demonstrated better feature extraction during MAE pretraining, as evidenced by more detailed reconstructions. However, this did not translate into improved segmentation performance, as the segmentation results exhibited more pronounced mosaic effects. This could be attributed to the increased number of parameters associated with smaller patches, requiring more training epochs to fully optimize.

Finally, the overall dice scores were not as high as anticipated across all datasets. This could be attributed to the inherent difficulty of the thyroid nodule segmentation task, which presents challenges due to the variable shapes and indistinct boundaries of the nodules. Future experiments could explore different training strategies or architectures to further enhance performance.

Beyond the technical metrics, our MAE-pretrained segmentation pipeline can be developed into a fully automated workflow that significantly reduces manual delineation by radiographers and radiologists—especially when processing large volumes or multiple nodules. Even DSCs in the 0.60–0.65 range can cut annotation time, improve consistency, and lower clinician workload compared to current FDA-approved semi-automated tools, which still require expert-drawn contours (Tessler and Thomas, 2023).

## 5 Conclusion

This study presents a transformer-based approach to thyroid nodule segmentation in ultrasound images, leveraging MAE pre-training to accelerate convergence and enhance feature learning. On three public datasets, our model achieved Dice Similarity Coefficients of 0.63 (AIMI), 0.64 (TN3K), and 0.65 (DDTI), demonstrating the feasibility of self-supervised pre-training even with limited annotated data.

Incorporating a cross-attention module did not yield consistent accuracy gains—likely because those layers were not pre-trained. Although smaller patch sizes improved reconstruction quality, they also introduced mosaic artifacts, increased context length, and added parameter complexity, resulting in longer convergence times without boosting segmentation performance.

Moving forward, we will integrate boundary-aware loss functions and adopt more extensive data-augmentation strategies to better delineate irregular nodule borders, and we will assemble larger, more diverse datasets to mitigate small-sample limitations. Even at moderate DSC levels, our MAE-driven auto-segmentation pipeline holds promise for reducing the manual delineation workload of radiographers and radiologists, thereby enabling more efficient and scalable clinical workflows.

## Data Availability

Publicly available datasets were analyzed in this study. This data can be found at: https://stanfordaimi.azurewebsites.net/datasets/a72f2b02-7b53-4c5d-963c-d7253220bfd5, https://www.kaggle.com/datasets/eiraoi/thyroidultrasound, and https://github.com/haifangong/TRFE-Net-for-thyroid-nodule-segmentation.
